# Supplementary material for: Evaluation of the Horizontal Transmission of White Spot Syndrome Virus for Whiteleg Shrimp (Litopenaeus vannamei) Based on the Disease Severity Grade and Viral Shedding Rate
Source: Animals (Basel). 2023 May 18;13(10):1676. doi: 10.3390/ani13101676 (PMC10215214; doi:10.3390/ani13101676)
Supplement: Supplementary file 1 [file animals-13-01676-s001.zip › Figure. S1.pdf]

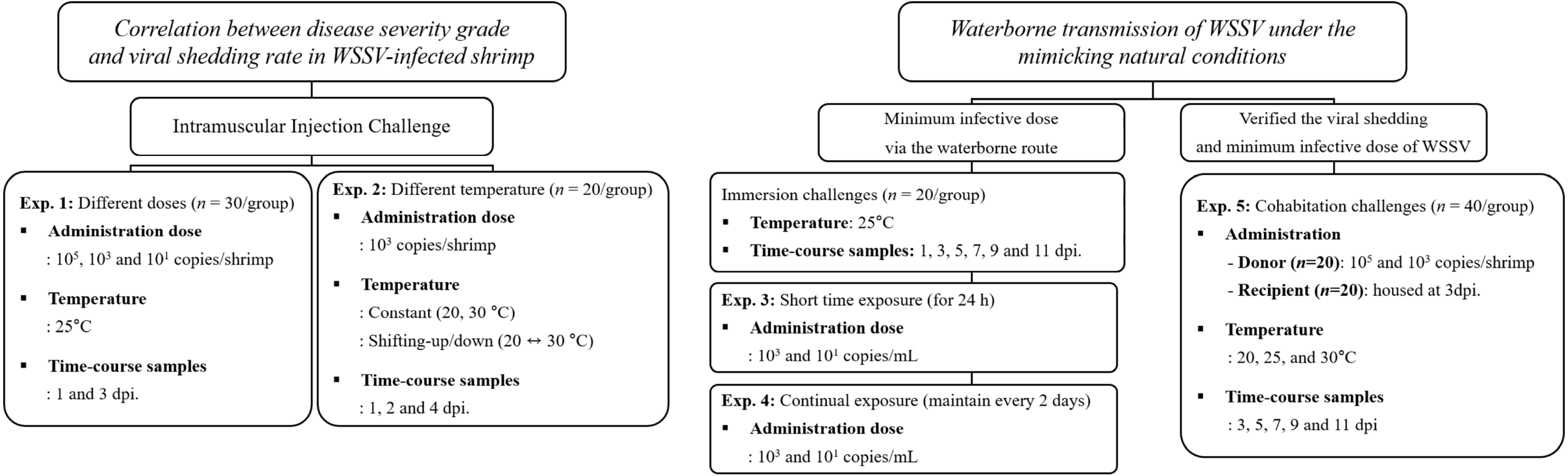
Correlation between disease severity grade and viral shedding rate in WSSV-infected shrimp
Intramuscular Injection Challenge
Exp. 1: Different doses (n = 30/group)
Administration dose
: 10^5, 10^3 and 10^1 copies/shrimp
Temperature
: 25°C
Time-course samples
: 1 and 3 dpi.
Exp. 2: Different temperature (n = 20/group)
Administration dose
: 10^3 copies/shrimp
Temperature
: Constant (20, 30 °C)
: Shifting-up/down (20 ↔ 30 °C)
Time-course samples
: 1, 2 and 4 dpi.
Waterborne transmission of WSSV under the mimicking natural conditions
Minimum infective dose via the waterborne route
Verified the viral shedding and minimum infective dose of WSSV
Immersion challenges (n = 20/group)
Temperature: 25°C
Time-course samples: 1, 3, 5, 7, 9 and 11 dpi.
Exp. 3: Short time exposure (for 24 h)
Administration dose
: 10^3 and 10^1 copies/mL
Exp. 4: Continual exposure (maintain every 2 days)
Administration dose
: 10^3 and 10^1 copies/mL
Exp. 5: Cohabitation challenges (n = 40/group)
Administration
- Donor (n=20): 10^5 and 10^3 copies/shrimp
- Recipient (n=20): housed at 3dpi.
Temperature
: 20, 25, and 30°C
Time-course samples
: 3, 5, 7, 9 and 11 dpi
