# Supplementary material for: Evaluation of the Horizontal Transmission of White Spot Syndrome Virus for Whiteleg Shrimp (Litopenaeus vannamei) Based on the Disease Severity Grade and Viral Shedding Rate
Source: Animals (Basel). 2023 May 18;13(10):1676. doi: 10.3390/ani13101676 (PMC10215214; doi:10.3390/ani13101676)
Supplement: Supplementary file 1 [file animals-13-01676-s001.zip › Table S1.pdf]

Table S1. Primer used in this study

Primer	Sequence(5'-3')	Purpose	Reference
146F1	ACT ACT AAC TTC AGC CTA TCT AG	WSSV detection	Lo et al. [22]
146R1	TAA TGC GGG TGT AAT GTT CTT ACG A		
146F2	GTA ACT GCC CCT TCC ATC TCC A		
146R2	TAC GGC AGC TGC TGC ACC TTG T		
WSS1011F	TGG TCC CGT CCT CAT CTC AG	WSSV detection and quantification	Durand and Lightner. [13]
WSS1079R	GCT GCC TTG CCG GAA ATT A		
TaqMan probe	AGC CAT GAA GAA TGC CGT CTA TCA CAC A		
VP 28 P1	AAG GAT CCG GAG AGC GTC ATG GAT CTT TCT TTC AC	Relative quantification	Jia et al. [25]
VP 28 P2	CCC CCC GAA TTC CAC GAT TTA TTT ACT CGG TCT C		
β-actin (F)	CCA CGA GAC CAC CTA CAA C		Anirudhan et al. [26]
β-actin (R)	AGC GAG GGC AGT GAT TTC		

References

13. Durand, S.V.; Lightner, D.V. Quantitative real time PCR for the measurement of white spot syndrome virus in shrimp. *J. Fish Dis.* **2002**, *25*, 381–389.
22. Lo, C.F.; Ho, C.H.; Peng, S.E.; Chen, C.H.; Hsu, H.C.; Chiu, Y.L.; Chang, C.F.; Liu, K.F.; Su, M.S.; Wang, C.H.; et al. White spot syndrome baculovirus (WSBV) detected in cultured and captured shrimp, crabs and other arthropods. *Dis. Aquat. Org.* **1996**, *27*, 215–225.
25. Jia, X.H.; Zhang, C.L.; Shi, D.J.; Zhuang, M.M.; Wang, X.; Jia, R.; Zhang, Z.Y.; Huang, J.; Sun, Y.H.; Qian, W.Y.; et al. Oral administration of Anabaenaexpressed VP28 for both drug and food against white spot syndrome virus in shrimp. *J. Appl. Psychol*. **2016**, *28*, 1001–1009.
26. Anirudhan, A.; Okomoda, V.T.; Iryani, M.T.M.; Andriani, Y.; Abd Wahid, M.E.; Tan, M.P.; Danish-Daniel, M.; Wong, L.L.; Tengku-Muhammad, T.S.; Mok, W.J.; et al. Pandanus tectorius fruit extract promotes Hsp70 accumulation, immune-related genes expression and Vibrio parahaemolyticus tolerance in the whiteleg shrimp Penaeus vannamei. *Fish Shellfish Immunol.* **2021**, *109*, 97–105.
